# SPaM: soft patch matching for non-rigid pointcloud registration

**DOI:** 10.3389/frobt.2023.1019579

**Published:** 2023-07-17

**Authors:** Behnam Maleki, Raphael Falque, Teresa Vidal-Calleja, Alen Alempijevic

**Affiliations:** Robotics Institute, University of Technology Sydney, Ultimo, NSW, Australia

**Keywords:** deformable registration, non-rigid registration, soft patches, patch matching, pointcloud registration, as rigid as possible

## Abstract

3d reconstruction of deformable objects in dynamic scenes forms the fundamental basis of many robotic applications. Existing mesh-based approaches compromise registration accuracy, and lose important details due to interpolation and smoothing. Additionally, existing non-rigid registration techniques struggle with unindexed points and disconnected manifolds. We propose a novel non-rigid registration framework for raw, unstructured, deformable point clouds purely based on geometric features. The global non-rigid deformation of an object is formulated as an aggregation of locally rigid transformations. The concept of locality is embodied in soft patches described by geometrical properties based on SHOT descriptor and its neighborhood. By considering the confidence score of pairwise association between soft patches of two scans (not necessarily consecutive), a computed similarity matrix serves as the seed to grow a correspondence graph which leverages rigidity terms defined in As-Rigid-As-Possible for pruning and optimization. Experiments on simulated and publicly available datasets demonstrate the capability of the proposed approach to cope with large deformations blended with numerous missing parts in the scan process.

## 1 Introduction

Given only point clouds, a common solution to the registration problem is to leverage mesh reconstruction, which is challenging for many depth sensors due to sensor noise, missing parts, and holes (stemming from occlusions). Mesh-based approaches [for example, leveraging Poisson surface reconstruction (Kazhdan et al., 2006)] are mainly suited to noise-free, water-tight surfaces. These methods generally involve interpolation and smoothing, consequently compromising the registration accuracy and potentially losing important details. Besides, other mesh reconstruction approaches (such as Ball Pivoting) leave some un-indexed points and disconnected manifolds, posing a challenge for state-of-the-art non-rigid registration techniques such as functional maps (Ovsjanikov et al., 2012).

If one were asked to manually determine the corresponding parts of two 3D point clouds (particularly of the challenging case of a large, featureless, semi-flat surface), one would start by taking the most conspicuous areas on one scan and look for correspondences on the other. By using these matched sections as the initialization step, the adjacent parts could be compared and evaluated in a transitive and progressive manner to grow a network of correspondences.

Following this idea, we propose a framework that is based on locally rigid patches and relies merely on geometrical features of 3D point clouds. Our proposed approachassumes that the aggregation of entire local rigidities accounts for the global non-rigidity of a deformable object throughout consecutive scans. Hence, we employ a concept of soft patches, whose primary benefit (compared with individual points), is a significant reduction of computational complexity and time.

**FIGURE 1:**
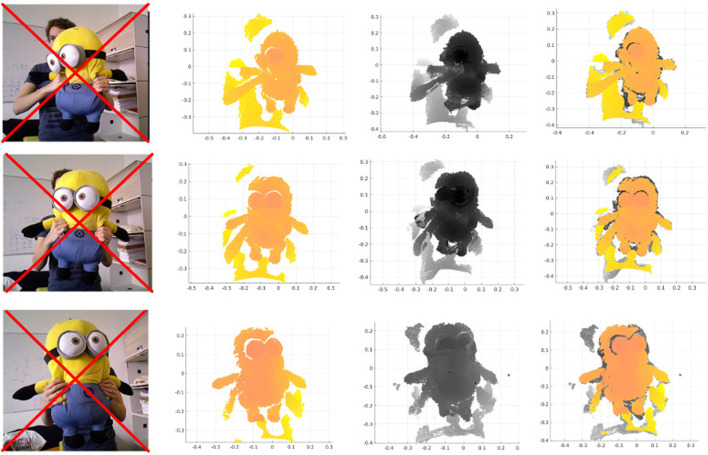
RGB image (not used), current frame, canonical frame and their registration for Minion dataset.

The main contribution of our work is a meshless, visually-featureless, model-free, topology-aware, and geometry focused approach for 3D non-rigid registration that enables the 3D reconstruction of deformable objects. Figure 1 shows an example of this registration on a public dataset. According to Cadena et al. (2016), our soft patch can be categorized as a low-level but flexible dense representation (with negligible computational overhead). In our pipeline, we obtain a sparse correspondence between (not necessarily consecutive) scans, which enables the approach to cope with small to large local deformations. We evaluate the proposed 3D registration method as well as the full reconstruction pipeline in simulated and publicly available datasets, demonstrating the validity of our approach.

## 2 Related work

The problem of registering successive belonging to deformable objects is often encountered in 3D mapping algorithms. Model or template-based methods for instance can handle severe deformation of an object quickly and effectively (Petit et al., 2017). The authors register a segmented point cloud in a rigid manner and then model elasticity by non-rigidly fitting a mesh based on the finite element method. In other approaches, an articulated motion model or additional proprioceptive sensors are used as priors to constrain and track the frame-to-frame non-rigid deformations to improve the reconstruction quality for fast body motion (Yu et al., 2017; 2018; Zheng et al., 2018). This reliance on other sensors or priors severely limits the applicability of these approaches.

Their surface representation based on Truncated Signed Distance Function (TSDF) is extracted by marching cubes and stored as a polygon mesh with point-normal pairs in the canonical frame. A single volume is registered to a single point in time (canonical frame), Dou et al. (2016) claim that the frame-to-frame motions in DynamicFusion are slow and carefully controlled due to the assumption on closest point correspondences between volume and frame. Thus, the approach cannot handle drastic deformation attributed to the challenges of fusing data back into a single model.

While DynamicFusion solely uses geometric correspondences, VolumeDeform (Innmann et al., 2016), upon generating a polygonal mesh, additionally takes advantage of RGB data via sparse globally consistent SIFT features to improve the alignment process. These features serve as global anchor points to mitigate drift and enable handling tangential motion. The methods relying on visual and colour features of the scene fail in the corresponding stage in the presence of visually featureless objects, poorly-illuminated scenes, or drastic changes of view.

The noted approaches generally use a mesh for establishing correspondences and extracting features for inter-frame motion tracking. However, surface reconstruction in the case of sparse and noisy point clouds is a cumbersome task. Further, the method proposed by Newcombe et al. (2015) and Innmann et al. (2016) may fail under larger inter-frame motion due to the underlying mesh-based correspondence estimation (Slavcheva et al., 2017).

The recent works on rigid and non-rigid registrations make extensive use of the Signed Distance Function (SDF) and its different variants such as TSDF, probabilistic SDF (PSDF), and Euclidean SDF (ESDF) (Gupta et al., 2016; Slavcheva et al., 2017; Yu et al., 2017). This body of work has proven to be effective volumetric representations of a scene. To address the sensor noise, they smooth out errors in cumulative models. Overall, the performance of these algorithms highly depends on the scenario, and the capability to handle the range and speed of deformation is always a compromise against fidelity. In contrast to existing dense SLAM approaches, the recent work SurfelWarp (Gao and Tedrake, 2019) enhances DynamicFusion by replacing volumetric data structures with surfel-based representation of geometry and a deformation field. In their pipeline for aligning the reference frame with the current one, they first initialize the deformation field from the previous frame and then similarly to DynamicFusion (Newcombe et al., 2015) deploy an iterative closest point (ICP) based optimization to estimate the non-rigid warp field.

The majority of the non-rigid registration approaches are taking advantage of the transformation regularization term in their cost function. However, in motion boundaries (discontinuities of the ground-truth optical flow between 2 frames), it may create artifacts. Addressing this issue, Zampogiannis et al. (2019) used two notions of contact and separation to annotate the changing topology of a scene and then blended two forward and (inverted) backward warp fields locally, according to the type and proximity of detected events. The warp fields are computed separately by non-rigid ICP, and the correspondence association is improved by the frame image.

## 3 Overview

Given two *oriented* unorganized point clouds of a deforming object captured in different timestamps, one is used as the target point cloud, P , and the second as the source, P . The objective is then to find the local rigid transformations, to register the associated soft patches from source, **C**, onto the corresponding soft patches of the target, **C**, to transform and gradually register the entire source point cloud onto the target.

**FIGURE 2:**
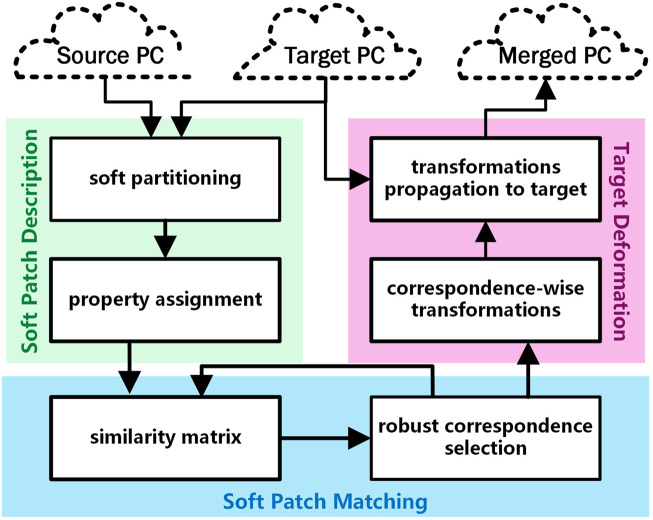
Flowchart of the devised framework.

The proposed framework includes three key constituents as describing the patches, evaluating the metrics of associations to establish the correspondence, and deforming the source locally. A flowchart schematic of our framework is depicted in Figure 2.

## 4 Soft patch description

### 4.1 Soft partitioning

Thus the definition of locality plays a pivotal role. Given two pointclouds from the same surface labelled as target and source which one has undertaken some deformation with respect to the other, the locality concept is then applied by independently subdividing these pointclouds into what we call partitions or patches. Let us assume that the corresponding partitions on the target and the source are rigidly transformed patches from one to the other. Then, it is possible to find the rigid transformation undergone by each patch individually, which consequently allows deforming the source non-rigidly to register with the target.

The aforementioned partitions have soft (in contrast to hard) boundaries, implying that each point in the pointcloud can belong to neighbouring patch (es) according to a measure called membership score. More specifically, the source and target are softly partitioned to create overlapping patches. Formally let us denote a pointcloud by $P=\left\{p_{1},\ldots ,p\right\}$ , such that the coordinate of the *i*th point is $p_{i}\in R^{3}$ and the number of points in P .

To differentiate between the target and the source properties, we make use of and superscripts, e.g., and denotes the number of points in target and source pointclouds, respectively. Note that multiple factors need to be considered while creating the partitions; the number of partitions, noted, should be proportional to the number of points in the pointcloud to generate meaningful patch descriptors. Also, the average number of points in patches, *q*, should be sufficient to create reliable local patch features as well as avoiding computational overhead. The other factor is the overlapping ratio of patches, so-called *softness* *τ*. Let C denote the set of overlapping patches $C=\left\{C_{1},\ldots ,C\right\}$ , then by following the aforementioned factors, the number of partitions for each pointcloud is computed by $≔\left\lceil /\left(q\times \left(1-\tau \right)+1\right)\right\rceil$ . The choice of the partitioning technique is irrelevant as long as the partitions are overlapping and the softness is controlled. In this work, we opt for k-medoids (Park and Jun 2009) with Euclidean distance, which is a variant of k-means with data points chosen as the centroids.

As a brief note in notation, throughout this work, the sequence of partition is denoted by centroids **M** = (**m**_*i*_|*i* = 1) and $M=\left(\left.m_{i}\right|i=1:\right)$ , where and correspond to the number of partitions in the target and the source, respectively.

### 4.2 Property assignment

Given the set of soft patches, the centroids are accompanied by a distance matrix **B**_×_ whose element *d*_*ij*_ is the Euclidean distance from **p**_*i*_ to the centroid of **C**_*j*_:

$$B_{\times }\;:\;=\left[\left.d_{ij}\right|d_{ij}={\left\|p_{i}-m_{j}\right\|}_{2}\;,\;i=1:\;,\;j=1:\right]$$ (1)

where ‖.‖_2_ denotes *L*_2_ norm. In order to determine whether a point *p*_*i*_ belongs to the soft patch *C*_*j*_, the term *d*_*ij*_ should be smaller than a threshold. To avoid having patches with largely varying sizes, we consider a single cut-off threshold for the distance, $\bar{d}$ . So, for each patch *C*_*j*_, we find the distance with which *q* points fall inside its boundary, and then computing the average of all these distances yields the above mentioned cut-off threshold. Thus, the patches are given by:

$$C_{j}=\left\{\left.p_{i}\right|{\left\|p_{i}-m_{j}\right\|}_{2}\leq \bar{d}\;,\;i\in \left\{1:\right\}\;,\;j\in \left\{1:\right\}\right\}.$$ (2)

To evaluate the similarity between 3D soft patches in different point clouds of the same surface, we use 3D SHOT descriptor (Salti et al., 2014), which produces a 352-tuple SHOT descriptor vector per point, **p**_*i*_. Let us define this descriptor vector of each point as *point SHOT*, **D**_*SH*_(**p**_*i*_). Then for each (soft) patch, **C**_*j*_, we define a so-called descriptor called *patch SHOT*, **D**_*CSH*_(**C**_*j*_), which is the normalised element-wise average of all including point SHOTS in **C**_*j*_:

$$D_{\mathrm{CSH}}\left(C_{j}\right):\;=\frac{\underset{p_{i}\in C_{j}}{\sum }D_{SH}\left(p_{i}\right)}{j}\times {\left\|\frac{\underset{p_{i}\in C_{j}}{\sum }D_{SH}\left(p_{i}\right)}{j}\right\|}_{2}^{-1}.$$ (3)

**FIGURE 3:**
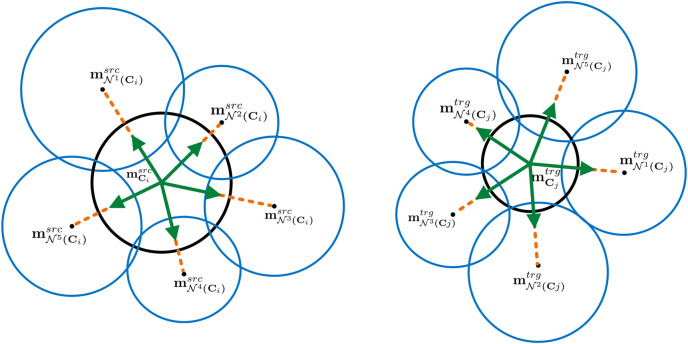
Schematic of a neighbourhood topological constraint of two patches (black circles) on source and target.

In this framework, the neighbourhood of every patch is a critical property that offers a reliable measure in the association stage. Hence, a complete neighbourhood map of every patch is generated, which consists of the index of adjacent patches, their related patch SHOTs, and the directional position of neighbours. The adjacent patches are defined by $N\left(C_{j}\right)≔\left\{C_{k}|\;\|m_{k}-m_{j}{\|}_{2}\leq 2\bar{d}\;,\;j\wedge k\in \left\{1:\right\}\right\}$ ,

To preserve the neighbourhood and increase the correspondence likelihood of adjacent patches of already matched patches, each patch is attributed with a topological constraint given its neighbourhood. We name this property *directional neighbourhood*, and define it as a set of 3D Euclidean unit vectors (illustrated in green in Figure 3) representing the direction of the lines connecting the centre of a query patch to the centres of its adjacent patches:

$$V_{C_{j}}≔\left\{\left.{\vec{v}}_{k}\right|{\vec{v}}_{k}=\frac{m_{N^{k}\left(C_{j}\right)}-m_{C_{j}}}{\|m_{N^{k}\left(C_{j}\right)}-m_{C_{j}}{\|}_{2}}\;,\;k=1:\right\}$$ (4)

where $N^{k}\left(C_{j}\right)$ is the *k*th element in the neighbors list of *C*_*j*_.

## 5 Soft patch matching

### 5.1 Patch similarity

We propose two types of metrics to measure the pairwise similarity of the soft patches **C**_*i*_ and **C**_*j*_. These two metrics aim to reflect the confidence on the patch association. The definitions of the metrics are as follows.

#### 5.1.1 SHOT vector distance

To measure the similarity of two patches SHOT descriptor vectors, **D**_*CSH*_(**C**_*i*_) and **D**_*CSH*_(**C**_*j*_), in addition to the original 352-D patch SHOT vector, we also use a 32-D Short SHOT (Seib and Paulus, 2018) vector. The Short SHOT is less dependent on the point normals and beneficial in the presence of noise. In our measure, the similarity obtained from the short SHOT variant, $D_{\mathrm{CSH}}^{s}\left(.\right)$ is combined with the one from the original Long SHOT, $D_{\mathrm{CSH}}^{l}\left(.\right)$ . Then, the SHOT Vector distance *d*_*SV*_, uses *L*_1_ and *L*_2_ norms of both variants:

$$\begin{array}{ll} d_{SV}\left(C_{i},C_{j}\right) & =\frac{1}{\beta_{l}+\beta_{s}}\underset{y\in \left\{l,s\right\}}{\sum }\frac{\beta_{y}}{\alpha_{1}+\alpha_{2}}\times \left(\alpha_{1}\|D_{\mathrm{CSH}}^{y}\left(C_{i}\right)\right.\\ & \;-D_{\mathrm{CSH}}^{y}\left(C_{j}\right){\|}_{1}+\alpha_{2}\|D_{\mathrm{CSH}}^{y}\left(C_{i}\right)\\ & \;\left.-\;D_{\mathrm{CSH}}^{y}\left(C_{j}\right){\|}_{2}\right) \end{array}$$ (5)

where *α*_1_ and *α*_2_ are the weights associated with *L*_1_ and *L*_2_ norms and also *β*_*l*_ and *β*_*s*_ denote the weights of Long and Short SHOT variants, respectively.

#### 5.1.2 Neighborhood-topology-preserving SHOT distance

In this metric, the goal is to measure the similarity between two patches in terms of their neighbours’ patch SHOT vector. Since even for two corresponding patches, the placements and sizes of adjacent patches are not necessarily similar, we need to evaluate the similarity of those neighbour patches, which are located in similar positions with respect to two query patches. For example, in Figure 3, the similarity measure between $N^{3}\left(C_{i}\right)$ and $N^{1}\left(C_{j}\right)$ , and also $N^{5}\left(C_{i}\right)$ and $N^{3}\left(C_{j}\right)$ should contribute prominently in the overall similarity of **C**_*i*_ and **C**_*j*_.

First, the pairwise angles between the normalised vectors in $V_{C_{i}}$ and $V_{C_{j}}$ are computed and stored into a matrix Θ_×_. Given a threshold *ν* as the maximum allowed angular difference between these vectors, the matrix is updated by Θ ← *ν* − Θ. Here, *ν* depends on the number of generated patches and the partitioning softness. After setting the negative elements of this matrix to zero, Θ is normalized between 0 and 1. Thus, the rate of co-direction of the vectors associated with $N^{m}\left(C_{i}\right)$ and $N^{n}\left(C_{j}\right)$ is indicated by 0 ≤ Θ(*m*, *n*) ≤ 1. Then, the indices and values of the non-zero elements (*m*, *n*, Θ(*m*, *n*)) of this matrix are used to compute the Neighborhood-Topology-Preserving SHOT distance, *d*_*NSV*_:

$$I=\left\{\left.\left(m,n\right)\right|\Theta \left(m,n\right)\neq 0,m\in \left\{1:\right\},n\in \left\{1:\right\}\right\}$$ (6)

$$\begin{array}{c} d_{\mathrm{NSV}}\left(C_{i},C_{j}\right)=\frac{\underset{\left(m,n\right)\in I}{\sum }d_{SV}\left(C_{i},C_{j}\right)\Theta \left(m,n\right)}{\underset{\left(m,n\right)\in I}{\sum }\Theta \left(m,n\right)} \end{array}$$ (7)

### 5.2 Similarity matrix

Using the distance functions defined in the previous section, we then build a similarity matrix, **S**, storing the pairwise similarity between the target patches and the source patches. As the SHOT vectors are normalized, the maximum possible value of *d*_*SV*_(., .) and *d*_*NSV*_(., .) is $\sqrt{2}$ . Therefore, the (*i*,*j*)^*th*^ element of **S** is defined as a measure of the similarity between **C**_*i*_ and **C**_*j*_ as follow:

$$s_{ij}=\sqrt{2}-\frac{\kappa_{1}d_{SV}\left(C_{i},C_{j}\right)+\kappa_{2}d_{\mathrm{NSV}}\left(C_{i},C_{j}\right)}{\kappa_{1}+\kappa_{2}}$$ (8)

where *κ*_1_ and *κ*_2_ are the weights of the aforementioned distances. Thus: $S={\left[s_{ij}\right]}_{\times }$ .

### 5.3 Robust correspondence selection

Given that the row and the column indices of **S** represent the index of the patches on the target and the source, respectively, in theory, the maximum element of a row (as the target patch index) should give the index of the corresponding patch on the source. However, due to the partial overlapping of pointclouds there are some target patches that lack correspondence, and also, such an approach would inevitably produce outliers. To obtain a more robust matching approach, we propose an assignment and rejection strategy that takes advantage of the rigidity terms defined in a reformulation of As-Rigid-As-Possible (ARAP) as the assignment error (Sorkine and Alexa, 2007). Our proposed strategy can be regarded as a discrete combinatorial search that attempts to minimize the piecewise rigidity between the target and the source.

Given two sets of the target and the source centroids as the representative of the soft patches, **M** and **M**, and also *n* pairwise correspondences, denoted by ${\left[x_{i}\right]}_{n\times 1}↔{\left[y_{i}\right]}_{n\times 1}$ and alternatively **X**↔**Y**, representing $C_{x_{i}}↔C_{y_{i}}$ (i.e., $m_{x_{i}}↔m_{y_{i}}$ ), let us reformulate ARAP in this way: We first build a graph by *n* cells centred at a target centroid, $m_{x_{i}}$ , and *k*_*A*_ vectors (edges), ${\left[E_{i}\right]}_{3\times k_{A}}$ , connecting the centroid to its *k*_*A*_ neighbors using an adjacency list (obtained by a k-d tree). Using this adjacency list and $m_{y_{i}}$ a graph (formed by *n* cells) is then built on the source yielding ${\left[E_{i}\right]}_{3\times k_{A}}$ (this process is illustrated in Figures 9C, D). We then compute the local rigidity term produced by the transformation of each cell from the target to the source. According to ARAP (Sorkine and Alexa, 2007), the optimal rotation **R**_*i*_ applied to the *i*th centroid **m**_*i*_ and its *k*_*A*_ neighbors $N\left(m_{i}\right)$ is obtained by using singular value decomposition (SVD):

$$\begin{array}{cc} & U_{i}\Sigma_{i}V_{i}^{*}\leftarrow \text{SVD}\left(\left[E_{i}\right]{\left[E_{i}\right]}^{⊺}\right)\\ & R_{i}=V_{i}U_{i}^{⊺} \end{array}$$ (9)

while enforcing that det (**R**_*i*_) > 0 by changing the sign of the column of **U** related to the smallest singular value.

Finally, the associated local rigidity error is obtained as:

$$r_{i}=\overset{k_{A}}{\underset{j=1}{\sum }}{\left\|E\left(:,j\right)-R_{i}E\left(:,j\right)\right\|}^{2}$$ (10)

and the global rigidity error is: $\sum_{i=1}^{n}r_{i}$ .

Thus, the selection can be formulated as a total rigidity minimization problem (implicitly local rigidity) induced by patch correspondence as:

$$\hat{G}\leftarrow \underset{X,Y,n}{\min}\frac{ARAP\left(X,Y\right)}{n}$$ (11)

subject to: *x*_*i*_ ∈ {1:}, *y*_*i*_ ∈ {1:}, *n* ∈ {1:} and if *i* ≠ *j* → *x*_*i*_ ≠ *x*_*j*_ ∧ *y*_*i*_ ≠ *y*_*j*_, where $\hat{G}≔{\left[\hat{X}\;\hat{Y}\right]}_{\hat{n}\times 2}$ denotes the established correspondences. This optimization is a special case of integer programming with an undetermined number of variables, i.e., *n*.

Due to the constraints and the unknown number of variables, we propose an optimization scheme. First, a batch of potential correspondences is achieved by associating each row of **S** to the column with the maximum element of the row. This batch is then filtered by iteratively rejecting the correspondence with the largest rigidity computed by ARAP, recomputing the ARAP for the remained batch and repeating these two steps until all local rigidities fall below a dynamic local rigidity upper-bound *u*_*l*_. After updating the similarity matrix (considering the established and the rejected correspondences), a new batch of potential correspondences is fed into the above filtering. This process terminates when the global rigidity of the established correspondences exceeds a dynamic total rigidity upper-bound, *u*_*t*_. These two upper bounds are first estimated in an initialization stage and then updated if no correspondence is established in the filtering stage, by setting *u*_*l*_ to the mean of the local rigidities associated with the currently established correspondences and *u*_*t*_ to the number of target patches times the median of the current established local rigidities.

This scheme is exhibited in Algorithm 1 as the function CorrespondingPatchSelection with three main stages: 1) a bootstrapping stage to initialize and propel the optimization by estimating the two upper-bounds obtained from *n*_0_ initial correspondences, 2) filtering the potential correspondences constrained by the current upper-bounds, 3) updating the two upper-bounds upon no new added correspondence.

Algorithm 1 Scheme for corresponding patch selection.

1: **function** CorrespondingPatchSelection(**S**)
2: [Δ]_×1_≔Max $\left(Di\mathrm{ff}\left(Sort\left({row}_{1:}\left(S\right)\right)\right)\right)$

3: *u*_*t*_ ← inf; [*r*_*i*_] ←∅;
4: **while** $u_{t}>\sum_{i=1}^{\hat{n}}r_{i}$ **do**
5: **if** *initialization* = **True** **then**
6: $\left(G_{a}^{\prime },S,u_{t},u_{l}\right)\leftarrow$ OptInit**S**, *k*_*A*_, *n*_0_
7: **S**← SimUpdater $S,G_{a}^{\prime },\Delta ,“dis^{\prime \prime },“rew^{\prime \prime }$

8: $\hat{G}\leftarrow G_{a}^{\prime };\;\;\;\;\;\;\;\;initialization=False$

9: **end if**
10: **G′** ← The row-column subscripts of the maximum elements of **S** per non-corresponded rows
11: $\left({\left[G_{a}^{\prime }\right]}_{n_{a}^{\prime }\times 2},\left[R_{i}^{\mathrm{t2s}}\right],\left[r_{i}\right],G_{r}^{\prime }\right)\leftarrow$ CorrFilter $G^{\prime },\hat{G},k_{A},u_{l}$

12: **S** ← SimUpdater $S,G_{r}^{\prime },\Delta ,“pen^{\prime \prime }$

13: **if** $n_{a}^{\prime }\neq 0$ **then**
14: $\hat{G}\leftarrow \hat{G}\cup G_{a}^{\prime }$

15: **S**← SimUpdater $S,G_{a}^{\prime },\Delta ,“dis^{\prime \prime },“rew^{\prime \prime }$

16: **else**
17: *u*_*l*_ ←max ([*r*_*i*_])
18: *u*_*t*_ ← Median ([*r*_*i*_])×
19: **end if**
20: **end while**
21: **return** ${\left[\hat{G}\right]}_{\hat{n}\times 2}$ and ${\left[R_{i}^{\mathrm{t2s}}\right]}_{3\times 3\times \hat{n}}$

22: **end function**

Given *n*_0_ as the initial number of correspondences in the bootstrapping stage, $\left\lceil \frac{n_{0}}{2}\right\rceil$ of them are acquired by the $\left\lceil \frac{n_{0}}{2}\right\rceil$ largest values of **S** as the most likely correspondences, and the remaining are associated with $n_{0}-\left\lceil \frac{n_{0}}{2}\right\rceil$ lowest local rigidities given by ARAP function fed by the first batch of potential correspondences (potential corresponding patches). Then the two upper-bounds are initialized as mentioned before. In Algorithm 1 this stage is defined as the function OptInit.

The foundation of our scheme is the potential-correspondence filtering (CorrFilter in Algorithm 1) which iteratively filters the potential input correspondences ${\left[G^{\prime }\right]}_{\left(-\hat{n}\right)\times 2}$ achieved as the row-column subscripts associated with the maximum value of the non-associated rows and then as the output, set some as accepted, $G_{a}^{\prime }$ , or rejected $G_{r}^{\prime }$ $\left(≔G^{\prime }\backslash G_{a}^{\prime }\right)$ . In this filtering process, given the already established correspondences, $\hat{G}$ , discarding the potential correspondence related to the highest local rigidity (obtained from the embedded *ARAP* function with *k*_*A*_ neighbours) continues until no rigidity is higher than *u*_*l*_.

Using a reward-penalty table, Δ, (acquired as the row-wise maximum value of the differentiated descendingly sorted rows in the original (un-updated) similarity matrix), **S** is updated by SimUpdater in Algorithm 1, in three ways denoted by three arguments *dis*, *rew* and *pen* as follows: 1) Disabling: in case of a newly added corresponding patch, the associated rows and columns are disabled by setting all elements to −inf. 2) Rewarding: when a correspondence is established, the elements of **S** associated with the co-directed neighbours are incremented by using Δ. 3) Penalizing: the rejected correspondence is penalized by decrementing their associated similarity score using Δ.

After updating **S**, the newly established correspondences are appended to $\hat{G}$ , and the adopted mechanism ensures no correct correspondences are falling below the current local upper bound. Then, upon no accepted correspondence, the two upper bounds are updated as there is a need to keep increasing the upper bounds to allow the correct but largely-deformed patches to be matched.

The optimization process terminates by reaching the global rigidity to the total upper bound.

## 6 Source warp

### 6.1 Correspondence-wise transformations

The useful by-product of the proposed optimization scheme (in Algorithm 1 is the centroid-wise target to source rotation, $R_{i}^{\mathrm{t2s}}$ , per correspondence *i*, whose inverse, $R_{i}^{\mathrm{s2t}}={\left(R_{i}^{\mathrm{t2s}}\right)}^{-1}$ , is the rotation required to transform the source centroid to the position of the corresponding target one. Using the position of the matched centroids, the associated translation is:

$$t_{i}^{\mathrm{s2t}}=m_{x_{i}}-R_{i}^{\mathrm{s2t}}m_{y_{i}}$$ (12)

with which the transformation matrix, $T_{i}^{\mathrm{s2t}}$ , to transform the *i*th matched source centroid to the corresponding target one is obtained by $T_{i}^{\mathrm{s2t}}=\left[\begin{array}{cc} R_{i}^{\mathrm{s2t}} & t_{i}^{\mathrm{s2t}}\\ 0_{1\times 3} & 1 \end{array}\right]$ .

### 6.2 Transformations propagation

By having these transformations, the points of the source pointcloud in the vicinity of a matched source centroid are impacted proportionally to their distance with respect to the centroid. Given the set of all matched source centroids, $M\left(\hat{Y}\right)=\left\{m_{{\hat{y}}_{i}}|{\hat{y}}_{i}\in \hat{Y}\right\}$ , the indices of *k*_*p*_ matched source centroids adjacent to a query source point **p**_*i*_ are shown by **A** (**p**_*i*_). So the *k*_*p*_ nearest centroids to point **p**_*i*_ are:

$$M\left(A\left(p_{i}\right)\right)=\left\{m_{a_{j}}|a_{j}\in A\left(p\right)\subset Y,\;j=1:k_{p}\right\}$$ (13)

where, $M_{A\left(p_{i}\right)}\subset M_{\hat{Y}}\subset M$ . As each point of the pointcloud may have several already matched centroids in its vicinity, it should be transformed based on the weighted interpolation of all those adjacent transformations. The weight, *w*, reflects the amount of influence is received from the adjacent matched centroids. According to Eberly (2017), the interpolation (loosely speaking the average) function for two transformations in **SE(3)** is a curve that follows the shortest geodesic path between two transformations. By extending their work to a weighted multi-transformation averaging, the transformation imposed on **p**_*i*_ is computed by, ${\bar{T}}_{p_{i}}={\text{Ave}}_{\mathrm{SE3}}\left(T_{p_{i}},W_{p_{i}}\right)$ , where $T_{p_{i}}=\left(T_{a_{j}}^{\mathrm{s2t}}|j=1:k_{p}\right)$ and $W_{p_{i}}=\left(w_{a_{j}}|j=1:k_{p}\right)$ . For the weights, we opted for Embedded Deformation weighting scheme (Sumner et al., 2007), where the weights of the *K* adjacent centroids to the point *i*, are computed by: $w_{a_{j}}=1-{\left\|p_{i}-m_{a_{j}}\right\|}_{2}^{2}/d_{\max}$ where *d*_max_ here is the distance to the *k*_*p*_ + 1 nearest centroid and then $w_{a_{j}}\leftarrow \frac{w_{a_{j}}}{\sum_{i=1}^{k_{p}}w_{a_{i}}}$ . Then, given ${\bar{T}}_{p_{i}}=\left[\begin{array}{cc} {\bar{R}}_{p_{i}} & {\bar{t}}_{p_{i}}\\ 0_{1\times 3} & 1 \end{array}\right]$ , the new position of source point *i* is computed as ${\tilde{p}}_{i}={\bar{R}}_{p_{i}}p_{i}+{\bar{t}}_{p_{i}}$ and the warped source to register on the target is $\tilde{P}={⋃}_{i=1}{\tilde{p}}_{i}$ .

## 7 Experimental results

In this section, we present the result of our experiments on two types of simulated and real datasets. To robustify the association, we used two concatenated SHOT descriptors (yielding a 704-D feature vector) per point, with big and small radii to account for the small and the large geometrical features. For the experiments in this section, the two SHOT radii, and *n*_0_ are set to 0.02 m, 0.05 m, and 10, respectively. Also, we set both *k*_*A*_ and *k*_*p*_ to 10.

### 7.1 Simulated-data

**FIGURE 4:**
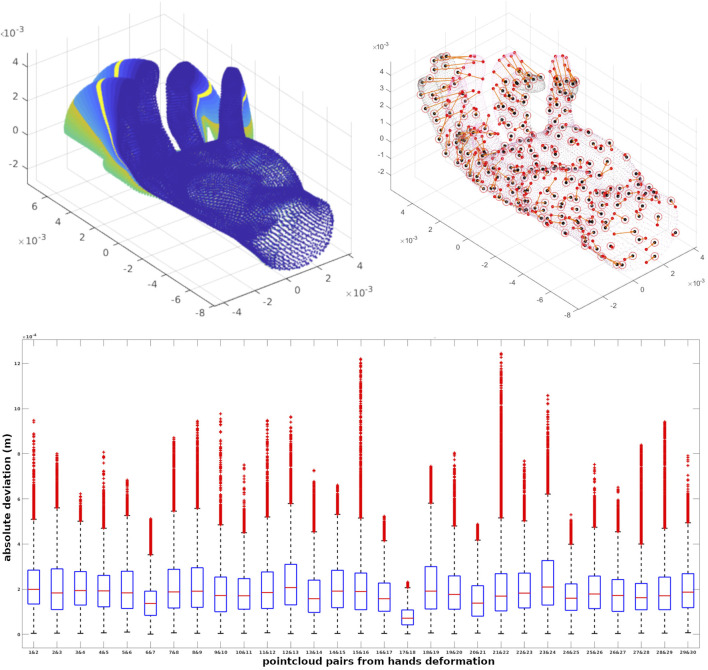
A quantitative evaluation is performed on 29 pairs of point clouds achieved from an animated 3D model. We show 30 different deformations of a hand, correspondences of articulation 1 and 7 depicted by centroids and the errors of points in registrations for 29 pairs of point clouds.

The quantitative evaluation of the framework is performed on an animated 3D model whose points are associated with a specific identifier code. As a result, each point representing a specific part of the surface shares the same index The frame-to-frame animation of the 3D model, shown in Figure 4, is performed with bounded biharmonic weights (Jacobson et al., 2011), which produces a smooth deformation. Figure 4A shows 30 different deformations of a hand generated through this method. By running our pipeline for 23894 points of this dataset, and choosing an average number of 100 points to be included in the partitions, in total 266 soft partitions for both source and target were generated. To show the robustness of the optimization, the detected correspondences of articulation 1, and articulation 7 of this dataset are depicted by centroids as representatives of patches in Figure 4B.

By warping all points of articulation *i* (source) to *i* + 1 (target) using the correspondences, there would be some deviation with respect to the actual position of the target points. The box plots associated with the errors of points in registrations for 29 pairs of point clouds are shown in Figure 4C. In these 29 experiments, the average number of the established correspondences is 210 out of 280 soft patches achieved in 100 iterations on average.

**FIGURE 5:**
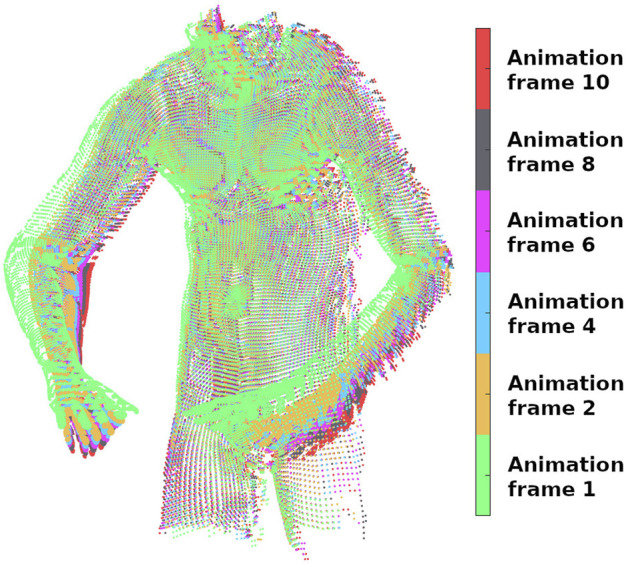
Six colour-coded animation frames of the human dataset captured by raytracing the associated original point clouds.

In another experiment with ground truth, we quantitatively compared the performance of our method against Zampogiannis et al. (2019), which is available as part of the Cilantro library (Zampogiannis et al., 2018). In this experiment, the goal is to create similar conditions as the capturing process with a single solid-state LIDAR camera (e.g., using a Realsense l515) in which the output point clouds are partially overlapping during deformation. For this experiment, we used the Deforming Human dataset with ground truth [generated again with bounded biharmonic weights (Jacobson et al., 2011)] in which each point of the surface has the same index in different animation frames. Simulating a fixed depth sensor by applying a raytracer algorithm (Skinner et al., 2014), the point clouds with different deformation were placed in front of the sensor iteratively, and the result of raytraced frames 1, 2, 4, 6, 8, and 10 are displayed in Figure 5 with the related colour-map for the frames. To make this experiment more realistic, sensor noise was simulated by applying zero-mean Gaussian noise with a standard deviation of 2 mm in the direction of the surface normal for each point.

**FIGURE 6:**
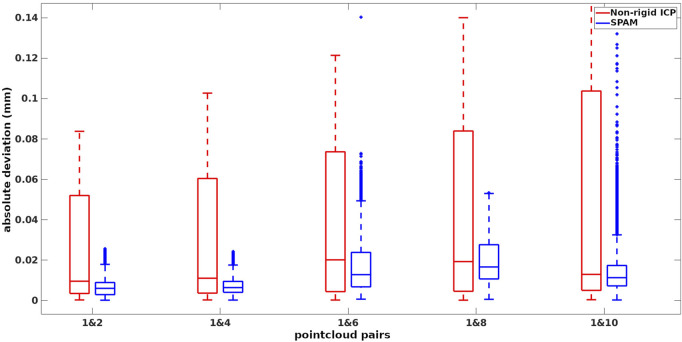
Deviations from ground-truth in 5 experiments from non-rigid ICP and SPaM on the selected pairs of deformation.

**FIGURE 7:**
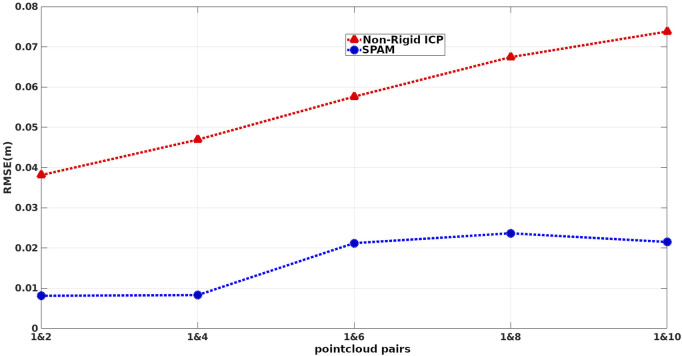
RMSE of distances between corresponding points of warped source and target on 5 sets of deformation.

During this process, the original indices (from the original articulation) of the raytraced points are saved. To evaluate the performance of methods in terms of handling different amounts of deformations, we paired the first (as the target) point cloud with the rest (as the source), giving 5 pairs of point clouds to be registered. Feeding these pairs into our pipeline and non-rigid ICP, we first found the corresponding points of the target and source (which share the same original indices) and then measured the absolute distance of those corresponding points in the warped source to the target. The box plot of deviations from ground truth associated with our pipeline (SPaM) and non-rigid ICP is illustrated in Figure 6, where the horizontal axis shows the experimental pairs, and the vertical axis represents the deviation from the ground truth in meters. In the box plots, the bottom and top edges of the box indicate the 25th and 75th percentiles, respectively; the central mark shows the median; and the outliers are represented by “+” symbol. The Root Mean Square Error (RMSE) acquired from these experiments are displayed in Figure 7.

### 7.2 Real-data

**FIGURE 8:**
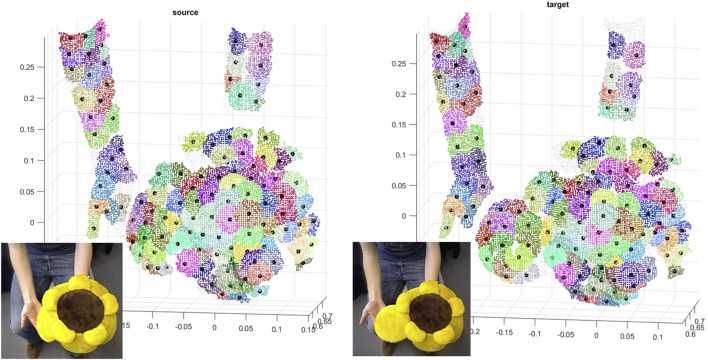
Color-coded patch-wise correspondences of soft patches on the source and the target including their centroids on Sunflower dataset.

We use the VolumeDeform dataset (Innmann et al., 2016), which contains a variety of deformable objects, to evaluate the performance of our method qualitatively. Our framework is capable of registering scans taken with a significant time difference, indicating the robustness of our optimization scheme for patch correspondence against large non-rigid local and global deformations. To demonstrate this capability, we used two pointclouds of the Sunflower dataset 30 frames apart. The scans are down-sampled and partitioned into soft patches (with 200 points on average), and then 95 corresponding patches were established (out of 125 and 139 soft patches of target and source). The soft partition concept is visualized in Figure 8.

**FIGURE 9:**
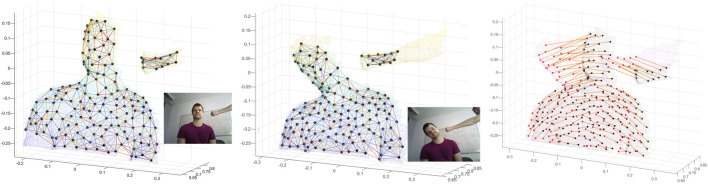
Registering two point clouds of the Boxing dataset, 40 frames apart.

We deployed our pipeline on boxing dataset as well; Figures 9A, B show the graph generated by the corresponding centroids and edges to 8 neighbour centroids (or rather patch centroids) for target and source. Figure 9D shows the target deformation onto the source by applying the average transformations of adjacent centroids to the points.

**FIGURE 10:**
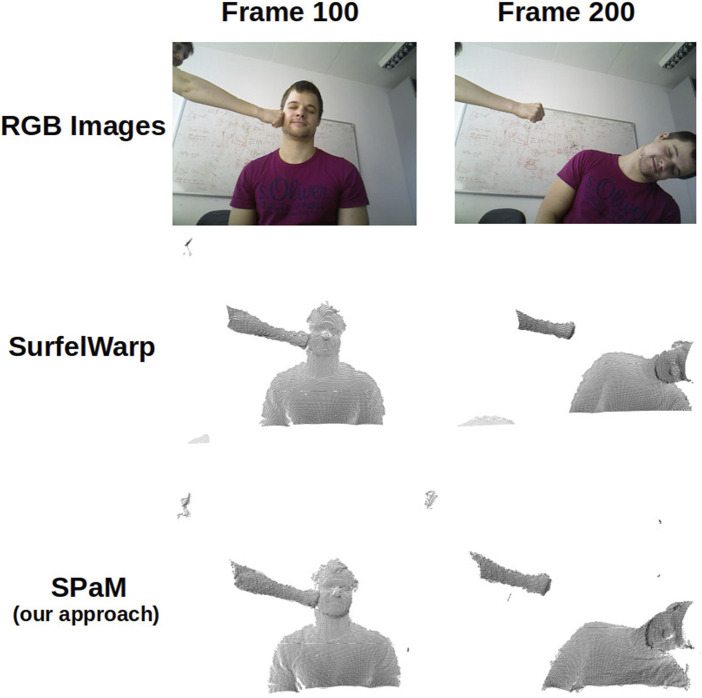
Qualitative comparison of our method (SPaM) compared to SurfelWrap (Gao and Tedrake, 2019) via the results of registration and reconstruction up to frames 100 and 200.

Although the focus of our proposed method is an offline CPU-based non-rigid registration (not reconstruction or fusion), we qualitatively compare the performance of SPaM on registering the consecutive frames with Surfelwarp (Gao and Tedrake, 2019). For this purpose, we use a simple iterative forward registration and fusion scheme. The current frame is regarded as the source and the merged model of all previous frames as targets (similar to a canonical frame). The objective in each iteration is, then, to register and warp the canonical frame towards the new frame. By using the boxing dataset, we merged the warped reconstructed model iteratively with the new scan. As there is not much deformation at the beginning of this dataset, we used frames from 40 to 200, and the result of reconstruction up to frames 100 and 200 acquired from two methods are compared in Figure 10, which shows the acceptable fidelity and alignment of the reconstructed model by SPaM to the current frame. It is worth mentioning that the average time for each CPU-based registration of this dataset is 120 s.

With the same conditions as above, we experimented on 170 consecutive scans of the minion dataset, and the results of three registrations are shown in Figure 1. The second column is the current frame, the third column is the reconstructed model, and the fourth column is the registration of the mentioned two pointclouds.

## 8 Conclusion

We have devised a framework (SPaM) to establish the correspondences of two non-rigidly deformed point clouds by using soft patches and an aggregation of locally rigid transformations. Our framework is evaluated on a challenging VolumeDeform dataset as capable of registering scans taken with a large time difference, indicating the robustness of our optimization scheme for patch correspondence against heavily non-rigid local and global deformations. However, on curved surfaces, the Euclidean distance is a suboptimal choice for propagating patch transformations to point elements. Re-organizing and optimizing our framework upon geodesic distance is a feasible option which we plan as an extension of our work. Also, by enhancing the optimization method, we plan to present this pipeline as a real-time approach.

## Data Availability

The datasets presented in this study can be found in online repositories. The names of the repository/repositories and accession number(s) can be found below: https://spamregistration.github.io/spamdataset/.
